# Vaccininum, Provings of

**Published:** 1883-05

**Authors:** B. Fincke

**Affiliations:** Brooklyn, N. Y.


### VACCININUM.

### Provings by B. Fincke, M. D., Brooklyn, N. Y.

1. Mary S., eight years, blonde, blue eyes, plump.

January 23d, 1862, 7½ A. M.—℞ Vaccinin, two globules, 1m (Fincke). After one hour: Aching and heat in whole head, especially forehead. Redness and distention of face, and chill running down back till afternoon.

January 24th.—Restless sleep. Severe pains in left upper arm at the vaccination mark, that she could not raise it, in the morning.

January 26th.—Eruption of small, red vesicles on left upper arm and chest, dying off after a few days.

*Remark:* This child was vaccinated on both arms when nine months old, and, on account of the health of the child and its parents, the lymph had been taken from the right arm for vaccination of many other children.

2. The same.

February 9th, 1866.—℞ Vaccinin, 2 gl., 85m (Fincke). Aching in forehead the whole forenoon; some belly-ache; stitch in left side of chest anteriorly under the short ribs.

3. The same.

April 30th, 1866.—℞ Vaccinin, 6 gl., 85m (Fincke). After a headache, which shortly disappeared, she felt uncommonly well.

4. Anna S., thirteen years, dark complexion; vaccinated, when an infant, on both arms with good effect.

January 16th, 1864, 4 P. M.—℞ Vaccinin, 21 gl., 1m (Fincke). (Small-pox prevalent in Washington and New York.) After a few hours: The forehead felt as if it would split in two in the median line from the root of nose to top of head, then stitches in right temple. Swelling of the neck under right ear (parotid gland), with sensation like being cut.

January 17th.—Slept well, but symptoms continued yesterday till evening, when they decreased.

January 18th.—Short breath, with aching in pit of stomach. In middle of night waked up by pain in forehead and eyes like split and stinging in both temples.

January 19th.—Puffed red face and red eyes, with small pimples on face and hands; splitting pain in forehead above nose; stinging in both temples; a stitch in hepatic region at margin of last lower rib, axillary line; some short breath, with pressure in region of heart; coffee tastes sour; no appetite; tongue coated, dryish yellow, with papillæ showing through coat; chill with shaking; crying; redness at upper arms, especially on left. The vaccination marks show no change. After lying down profuse perspiration broke out, after which chill, redness, and pimples disappeared.

January 20th.—The same splitting pain in forehead holds on yet; stitch at last lower ribs in splenic region.

January 21st.—Very weak.

January 22d.—Splitting pain in forehead still present; tearing in left thigh downward.

5. Mrs. S., about forty years, dark blonde, gray eyes, widow. Complained of weak eyes, falling out in the forehead like split.

February 20th—℞ Vaccinin, 1 gl., 1m (Fincke). After half an hour improvement, especially in eyes. After two hours: Stitches in right side under short ribs in front from right to left and then at corresponding place in left side, but from left to right, lasting five minutes. It was felt distinctly, not in the ribs but deeper inward, in liver and spleen. The splitting pain in forehead was removed, but not the symptoms of falling out.

6. An infant received a dose of Vaccinin 1m or higher. After awhile a pustule filled with matter, and, with a depression in the centre, appeared on the left shoulder and ran its natural course of desiccation.

The parents, not trusting to this mode of vaccination, had the child vaccinated in the ordinary way about a year after, when it took; and no wonder, for a great quantity of poison was put into the arm.

The opinion that when after the internal administration of Vaccinin in potency the inoculation with the crude virus is effective the insufficiency of vaccination by internal medication is proved is fallacious, because small-pox is not taken by inoculation, but in an unknown, insensible manner, which the internal mode of prevention may nevertheless be potent enough to counteract. Just so you might give a high potency of Hydrocyanic acid for prevention of the fatal effects of a drop of the crude acid afterward given, and expect that death would be prevented by such prophylaxis. The fallacy is palpable.

7. Ernest S., a healthy boy, four years.

April 30th, 1869.—Was vaccinated with an old scab, well preserved in wax.

May 10th.—A small pustule, with depression in centre and red halo developed on left shoulder. The inoculation scratch healed up with an indifferent scab. I took matter from the pustule on the seventh day and vaccinated other children, but it did not take.

For experiment's sake I took some of the scab, which I had the boy vaccinated with, dissolved in a little water, and rubbed it with my finger in his upper arm without making a wound.

May 13th.—The third day after this fever set in, and an eruption all over the body appeared of small pustules, some with a central depression, some brown. One on the left shoulder is nearly as large as the first one after inoculation. Later it grew larger and formed a good scab. Likewise the others desiccated in due time.

8. Miss F., twenty-six years, dark hair, brown eyes, gracile, good vaccination-marks at left upper arm.

January 20th, 1865.—℞ Vaccinin, 12 gl., 1m (Fincke).

February 3d.—Eruption of pustules with a dark red base and a roundish or oblong elevation filled with pus of a greenish yellow color, at left side of trunk, between shoulders, on left shoulder, behind right ear, resembling varioloid, some as large as a pea, some less, without depression in the centre, coming with a round, hard feel in skin (like a shot) very itchy, with nervous depression, impatience, irritability, disposition to be troubled by things, which is unusual with her. With all that, good appetite. Now, at this stage (small-pox prevailing) she was inoculated at the same left upper arm, where she has the good vaccination-marks, with matter derived from a scab dissolved in water, by one scratch. The scab was fresh and came from an apparently healthy infant.

February 4th.—Twelve hours after vaccination the pustules brought out by Vaccinin 1m (Fincke) were suddenly much depressed; the matter contained in them seemed to be reabsorbed in a great measure; they do not itch any more. At the same time she got sick, feverish, with twisting pains in both knees and in lower back; soreness in inner corner of left eye; confusion, she does not remember things at the time she wants them; gauzy sensation before eyes in morning, cannot see well.

February 10th.—The vaccination by arm did not proceed any further, and the scratch was healing up without forming a scab or scar.

In 1870, when in Paris, very much fatigued by the voyage and sightseeing, she lived over a room where her landlady died of small-pox at that time, but she was not affected.

In 1857 she took care of her little sister, who had varioloid, and slept with her, but did not take it.

9. Miss J. C., twenty years.

January 20th, 1865.—℞ Vaccinin, 6 gl., 1m (Fincke). Got some pimples in face.

February 3d.—Vaccinated with the scab used in the former case. Did not take.

10. Miss N., thirty years, single, dark blonde, blue eyes, muscular.

January 15th, 1870.—℞ Vaccinin 4 gl., 85m (Fincke). Severe headache all over, thought to be produced by excitement; relieved by Nux vom. 94m (Fincke); then she received another dose of Vaccinin 85m (Fincke) after the headache was over. About a week later the same headache returned again, passing off after awhile by itself. She had been vaccinated several times without effect.

11. Another lady, tall, dark complexion, about twenty years, single.

After one dose of Vaccinin 85m (Fincke): Weakness in small of back coming on suddenly when walking in Broadway; went home and to bed and it then gradually passed off.

12. Mrs. C. F., fifty-one years.

March 28th, 1871.—Took the vial of Vaccinin 30 (Fincke), in her left hand at 10.09 A. M. After four minutes: Prickling in left fourth finger, as if going to sleep; the vial remains cold in the warm hand; drawing at left elbow, up the arm; the same prickling as above in left temple, also some in left middle finger; left hand begins to feel hot; slight drawing up of left elbow; peculiar, dull sensation at left lower lid. 10.15 A. M., drawing at left elbow more painful. 10.16, left fourth finger begins to burn; a warmth streams through left arm; coolness at throat, anteriorly down the breastbone in and outwardly; slight tearing from left wrist into forepart of radius anteriorly; aching of left fourth finger; sensation as if heat passes from dorsum of hand, as of steam; aching at heart; drawing down left side of chest and in back; left upper arm feels stiff, feels somewhat tremulous. 10.22 A. M., shaking chill. The following night she could hardly get warm.

